# Trends and determinants of underweight and overweight/obesity among urban Ethiopian women from 2000 to 2016

**DOI:** 10.1186/s12889-020-09345-6

**Published:** 2020-08-24

**Authors:** Kedir Y. Ahmed, Solomon Abrha, Andrew Page, Amit Arora, Solomon Shiferaw, Fentaw Tadese, Canaan Negash Seifu, Tebikew Yeneabat, Emana Alemu, Delelegn Yilma Gebremichael, Abdulaziz Seiko, Felix Akpojene Ogbo

**Affiliations:** 1 Translational Health Research Institute, Western Sydney University, Campbelltown Campus, Campbelltown, Sydney, NSW Australia; 2 College of Medicine and Health Sciences, Samara University, Samara-Logia, Ethiopia; 3 School of Public Health, College of Medicine and Health Sciences, Wolayta Sodo University, Wolayta Sodo, Ethiopia; 4 School of Health Sciences, Western Sydney University, Campbelltown Campus, Campbelltown, Sydney, NSW Australia; 5 Oral Health Services, Sydney Local Health District and Sydney Dental Hospital, NSW Health, Surry Hills, Sydney, NSW Australia; 6 Discipline of Child and Adolescent Health, Faculty of Medicine and Health, Sydney Medical School, The University of Sydney, Westmead, Sydney, NSW Australia; 7 School of Public Health, College of Medicine and Health Sciences, Addis Ababa University, Addis Ababa, Ethiopia; 8 College of Medicine and Health Sciences, School of Public Health, Wollo University, Dessie, Ethiopia; 9 School of Nursing and Midwifery, Western Sydney University, Campbelltown Campus, Campbelltown, Sydney, NSW Australia; 10 Faculty of Health, University of Technology Sydney, Ultimo, Sydney, NSW Australia; 11 Ethiopian Public Health Institute, Addis Ababa, Ethiopia; 12 Department of Public Health, College of Medicine and Health Sciences, Ambo University, Ambo, Ethiopia; 13 CARE Ethiopia, Partner for The Resilience Project, Afar, Samara-Logia, Ethiopia; 14 General Practice Unit, Prescot Specialist Medical Centre, Makurdi, Benue State Nigeria

**Keywords:** Double burden of malnutrition, Underweight, Overweight, Obesity, Urban women, Ethiopia

## Abstract

**Background:**

Nutritional, epidemiological and demographic transitions have been associated with the emergence of the double burden of malnutrition globally. In Ethiopia, there has been no nationally representative investigation of trends and determinants of both underweight and overweight/obesity among urban women. This study examined the trends and determinants of underweight and overweight/obesity in urban Ethiopian women from 2000 to 2016.

**Methods:**

Trends in the prevalence of underweight and overweight/obesity were investigated based on a series of the Ethiopia Demographic and Health Survey (EDHS) data for the years 2000 (*n* = 2559), 2005 (*n* = 1112), 2011 (*n* = 3569), and 2016 (*n* = 3106). Multivariable multinomial logistic regression was used to investigate the association between socioeconomic, demographic, behavioural, and community-level factors with underweight and overweight/obesity.

**Results:**

The prevalence of underweight in urban Ethiopian women reduced significantly from 23.2% (95% confidence interval [CI]: 20.3, 26.3%) in 2000 to 14.8% (95% CI: 13.1, 16.7%) in 2016, while overweight/obesity increased significantly from 10.9% (95% CI: 9.1, 13.0%) in 2000 to 21.4% (95% CI: 18.2, 25.1%) in 2016. Urban women from rich households and those who had never married were less likely to be underweight. Urban women who were from wealthy households and those who attained at least secondary education were more likely to be overweight/obese. Women who were informally employed and listened to the radio were less likely to be overweight/obese compared to those who were unemployed and did not listen to the radio, respectively.

**Conclusion:**

The prevalence of overweight/obesity increased from 2000 to 2016, with a concurrent reduction in the prevalence of underweight. Interventions aiming to reduce overweight and obesity should target urban women with higher education, those who resided in wealthier households and those who watched the television.

## Background

Nutritional, epidemiological and demographic transitions have been associated with the emergence of the double burden of malnutrition worldwide [1–3]. The World Health Organization (WHO) defines the double burden of malnutrition as “the coexistence of undernutrition along with overweight, obesity or diet-related non-communicable diseases (NCDs), within individuals, households, and populations, and across the life-course” [4]. Malnutrition (e.g., underweight and overweight/obesity) is associated with short- and long-term adverse consequences [5]. In early pubertal women, overweight/obesity is associated with psychosocial problems and abnormal uterine bleeding due to irregularity in the menstrual cycle from peripheral conversion of androgens to oestrogen [6–8]. For older women, overweight/obesity is associated with an increased risk of gestational diabetes and pre-eclampsia, haemorrhage, caesarean birthing, and maternal and infant death during childbirth [9, 10]. In all populations, overweight/obesity is associated with an increased risk of NCDs such as Type 2 diabetes mellitus, cardiovascular and respiratory diseases [11].

Preventing malnutrition is one of the greatest global public health challenges as a result of a complex and non-linear relationship between nutritional, demographic and epidemiological transitions [4, 12]. Globally, nearly one-third of the population is affected by at least one form of malnutrition (either underweight or overweight/obesity) [4, 13]. In 2016, more than 600 million adults were underweight, while nearly 2 billion were overweight/obese [14–16]. Evidence has shown that both underweight and overweight/obesity are higher in women compared to men [17–19]. In low- and middle-income countries (LMICs, including Ethiopia), an increase in overweight/obesity prevalence has occurred alongside the reduction in the burden of underweight, particularly in women of reproductive age group [4, 13]. The increasing burden of overweight/obesity has been attributed to a range of factors, including micro- and macro-economic growth and urbanisation [20, 21].

In Ethiopia, a sub-national study showed a reduction in the proportion of urban women with underweight [22]. Similarly, a previous national study conducted in Ethiopia based on the 2011 Ethiopia Demographic and Health Survey (EDHS) data suggested that reproductive-aged women who were older, educated, married and those who resided in wealthier households were more likely to be overweight or obese compared to their counterparts [23]. Although important, these studies have several limitations. First, the studies did not consider the most recent national data (2016 EDHS). Up-to-date information based on the most recent data is essential as this survey potentially represents the current socio-demographic and economic context of the country. Second, these studies did not investigate the national trends in the prevalence of underweight and overweight/obesity as these data can provide additional information into where progress has been made and/or where specific efforts may be required. Third, these studies did not account for confounders in the modelling, a key methodological step in assessing an association between two variables of interest [24].

Understanding the national trends and determinants of underweight and overweight/obesity among women residing in urban households can inform policy responses towards the control and prevention of malnutrition in Ethiopia. This information is particularly useful to national and international stakeholders given the current implementation of nutrition efforts in Ethiopia [25, 26] within the context of the United Nation’s Sustainable Development Goal 2.2 (SDG–2.2, end all forms of malnutrition by 2030) [27] and the Global Action Plan for the Prevention and Control of NCDs target 9 (halt the rise in obesity) [28]. Accordingly, the present study aimed to investigate the trends and determinants of underweight and overweight/obesity in urban Ethiopian women from 2000 to 2016.

## Methods

### Data sources

This study used the Ethiopia Demographic and Health Survey (EDHS) data for the years 2000 (*n* = 2559), 2005 (*n* = 1112), 2011 (*n* = 3569), and 2016 (*n* = 3106). The data were collected by the Central Statistical Agency (CSA) and Inner City Fund (ICF) International, with funding from the United States Agency for International Development [29] and the Government of Ethiopia [30–33]. The EDHS used a two-stage stratified cluster sampling technique to select the study participants. In stage one, after each administrative region was stratified into urban and rural strata, Enumeration Areas (EAs) were selected using a probability proportional to EA size. In stage two, a household listing operation was carried out in all of the selected EAs and a fixed number of households from each EA were selected [30–33]. All women aged 15–49 years who were permanent residents or who spend the night in the selected households the night before the survey were included in the surveys [30–35]. A weighted total sample of 10,346 women was used, with high response rates that ranged from 94.6 to 97.8%. Detailed methodological strategies used in the surveys have been described elsewhere [30–33]. The present study focused on urban women because past studies have shown that urbanisation is a contributor to the double of malnutrition [20, 21], and women are more likely to be underweight and/or overweight/obese compared to men [14].

### Outcome variables

The main outcome variables were underweight and overweight/obesity, measured based on WHO adult body mass index (BMI) classification [15] and used by the Ethiopia Central Statistical Agency and ICF International [29]. BMI was defined as a woman’s weight in kilograms divided by the square of her height in meters (kg/m^2^). The EDHS used lightweight SECA mother scale to measure weight and Shorr measuring board to assess height [30–33]. BMI was classified into three groups:

- Underweight: BMI < 18.5 kg/m^2^
- Normal: BMI ≥ 18.5 kg/m^2^ and BMI ≤ 24.9 kg/m^2^
- Overweight/obesity: BMI ≥ 25.0 kg/m^2^

### Study variables

The study broadly categorised the study factors as socioeconomic, demographic, behavioural and community-level factors based on previous studies [36, 37]. The selected study factors are associated with underweight and overweight/obesity in reproductive-aged women in previously published studies from LMICs [22, 23, 38–41].

Socioeconomic factors included women’s highest education, women’s employment status, marital status, and household wealth status. Women’s education was classified as ‘no schooling’, ‘primary education or ‘secondary or higher education’. Women’s employment was classified as ‘no employment’, ‘formal employment’ (i.e., professional, technical, managerial, clerical, and services area workers), or ‘informal employment’ (i.e., agricultural and manual workers) [36, 42]. Marital status was classified as ‘never married’, ‘formerly married’ or ‘currently married’. The EDHS used principal components analysis (PCA) to calculate the household wealth index based on a series of variables relating to ownership of household assets such as television and bicycles; type of materials used for housing construction; and types of water source and sanitation facilities [43]. The household wealth index was classified as ‘poor’, ‘middle’ or ‘rich’, consistent with previously published studies [44, 45].

Demographic and behavioural factors included women’s age, parity, listening to the radio, reading newspapers/magazine, and watching television. Women’s age was classified as ‘15–24 years’, ‘25–34 years’ or ‘35 and above years’, and women’s parity classified as ‘none’, ‘1–4 children’ or ‘5 or more children’. Women who reported exposure to the media (radio, magazine/newspaper or television) at least once a week were classified as ‘Yes’ and those who did not were classified as ‘No’. Community-level factor (i.e. region of residence) was classified as ‘Tigray’, ‘Afar’, ‘Amhara’, ‘Oromia’, ‘Somali’, ‘Benishangul’, ‘Southern Nations Nationalities and Peoples Region (SNNPR)’, ‘Gambella’, or ‘Metropolis’ regions based on Ethiopia’s geopolitical and administrative features, consistent with the EDHS report and previously published studies [30–33, 36]. The Metropolis region included Addis Ababa and Dire Dawa city administrations, and the Harari region. Among the study participants, about 44.1% of women had no employment, and nearly half (47.1%) of them were in the 15–24 years’ age group (Additional file 1).

### Statistical analysis

Preliminary analyses involved the description of the study participants by calculating frequencies and percentages of the study variables. This was followed by the estimation of the prevalence of the outcome variables (underweight and overweight/obesity) and by the selected study variables (socioeconomic, demographic, behavioural and community-level factors) in both year-specific data (2000, 2005, 2011 and 2016) and in the combined dataset. Then, percentage point change with corresponding 95% CI of the outcome variables calculated by each of the study factors to examine the changes over the EDHS years (from 2000 to 2005, from 2005 to 2011, from 2011 to 2016 and from 2005 to 2016) [Additional files 2, 3 and 4]. We used the combined dataset to increase the statistical power of the study in order to detect any association between the study factors and the outcomes, as well as to examine trends in underweight and overweight/obesity over the study period (2000–2016).

Multivariable multinomial logistic regression modelling was used to examine the association between socioeconomic, demographic, behavioural and community-level factors and (i) underweight and (ii) overweight/obesity using the normal weight group as a reference category. Specifically, socioeconomic factors were entered into the model to assess their relationship with the outcomes, with adjustment for demographic, behavioural and community-level factors *(stage 1)*. A similar strategy was used in models of demographic factors to examine their relationship with the outcome variables, with additional adjustment for socioeconomic, behavioural and community-level factors *(stage 2)*. Similar modelling techniques were used for the behavioural and community-level factors in the third and fourth stages *(stages 3 and 4)*, respectively.

In the models, we adjusted for the survey years in the combined dataset, while sampling weight and clustering were accounted for in both the year-specific and combined datasets. Collinearity was checked using ‘variance inflation factor (VIF)’ but no significant results were evident in the analyses. We also estimated P for trends in each category of the study variables to assess for any convergence or divergence. Adjusted odds ratios with 95% confidence intervals (CIs) were estimated as the measure of association between study factors and outcome variables. All statistical analyses were conducted using Stata version 14.0 with ‘svy’ command to adjust for sampling weights, clustering effects and stratification, and the ‘mlogit’ function was used for the modelling.

## Results

### Prevalence of underweight and overweight/obesity

Over the study period (2000–2016), the highest prevalence of underweight was observed among urban women who resided in the Gambella region of Ethiopia (28.7%), followed by women from the Tigray region (28.0%). The lowest underweight prevalence was found among women from the SNNPR region (13.1%) (Table 1). During the same period, urban women aged 35–49 years had the highest prevalence of overweight/obesity (27.4%), followed by women who resided in the Somali region (23.0%). The lowest prevalence of overweight/obesity was observed among urban women aged 15–24 years of age (7.9%) (Table 2).

**Table 1:** Prevalence of underweight by study variables among urban women in Ethiopia, 2000–2016

Variables	2000	2005	2011	2016	2000–2016	2000–2016
	n (%)	n (%)	n (%)	n (%)	n (%)	^**a**^Diff (95% CI)
**Socioeconomic factors**						
Women’s education						
No schooling	230 (26.4)	55 (21.4)	155 (20.3)	67 (12.9)	507 (21.0)	−13.5 (−20.1, − 6.8)
Primary school	121 (19.3)	55 (19.5)	344 (22.4)	134 (12.7)	653 (18.7)	−6.6 (− 11.7, − 1.5)
Secondary and higher	242 (22.8)	99 (17.3)	217 (17.1)	259 (16.8)	816 (18.4)	−6.0 (−10.8, − 1.1)
Women’s employment						
No employment	278 (25.0)	123 (19.3)	318 (21.2)	226 (17.6)	946 (20.8)	−7.4 (−13.1, − 1.6)
Formal employment	176 (19.0)	58 (16.4)	260 (18.1)	183 (12.7)	677 (16.3)	−6.3 (−11.5, − 1.1)
Informal employment	129 (26.2)	28 (23.5)	131 (21.5)	50 (13.2)	338 (21.1)	−13.0 (−22.3, − 3.6)
Marital status						
Not married	256 (23.6)	113 (21.8)	354 (23.2)	246 (19.3)	970 (22.0)	−4.4 (−9.1, 0.5)
Currently married	204 (20.5)	53 (13.1)	260 (16.5)	132 (9.4)	650 (14.8)	−11.1 (−15.3, −6.8)
Formerly married	132 (27.9)	43 (22.6)	101 (21.5)	81,919.3)	357 (23.0)	−8.7 (−16.5, −0.6)
Household wealth status						
Poor	427 (26.5)	138 (22.4)	48 (51.0)	14 (12.5)	628 (25.7)	−11.3 (−22.4, −0.2)
Middle	121 (18.8)	42 (16.4)	4 (10.7)	14 (34.8)	181 (18.5)	16.3 (−9.3, 42.0)
Rich	4 (6.3)	11 (10.1)	716 (19.3)	431 (14.6)	1110 (16.9)	−9.9 (−13.3, −6.5)
Toilet facility						
Unimproved	196 (26.4)	115 (22.1)	477 (23.2)	226 (14.9)	1014 (21.0)	−11.6 (−18.0, − 5.2)
Improved	397 (21.8)	87 (15.4)	235 (15.8)	227 (14.8)	947 (17.5)	−7.0 (−11.8, −2.3)
Source of drinking water						
Unimproved	89 (24.5)	18 (21.1)	65 (21.3)	85 (15.8)	257 (19.9)	−8.7 (−15.3, −2.1)
Improved	504 (23.00	192 (18.6)	651 (19.9)	374 (14.6)	1719 (19.0)	−8.4 (−12.5, −4.3)
**Demographic factors**						
Women’s age						
15–24 years	291 (23.2)	114 (20.1)	385 (22.2)	255 (19.4)	1045 (21.5)	−3.8 (−8.3, 0.7)
25–34 years	127 (18.5)	53 (18.6)	190 (17.3)	124 (11.7)	494 (15.8)	−6.8 (− 11.9, − 1.7)
35–49 years	174 (28.4)	41 (16.1)	141 (19.0)	80 (10.9)	437 (18.6)	−17.5 (−24.1, − 10.9)
Parity						
None	285 (23.0)	120 (20.6)	382 (22.1)	288 (18.9)	1074 (21.2)	−4.1 (−8.5, 0.4)
1–4 children	188 (21.4)	59 (15.3)	245 (16.8)	144 (11.0)	636 (15.8)	−10.4 (−15.2, −5.6)
5+ children	120 (27.1)	29 (21.1)	90 (23.3)	28 (9.9)	267 (21.4)	−17.3 (−25.7, −8.8)
**Behavioural factors**						
Listening radio						
No	165 (26.2)	44 (20.9)	216 (25.5)	228 (16.8)	653 (21.5)	−9.4 (−15.3, −3.5)
Yes	427 (22.2)	165 (18.4)	499 (18.4)	231 (13.2)	1322 (18.1)	−9.0 (−13.2, −4.8)
Read magazine						
No	354 (22.9)	105 (19.9)	411 (20.9)	295 (14.2)	1165 (19.1)	−8.7 (−12.9, −4.6)
Yes	239 (23.5)	102 (17.7)	304 (19.2)	164 (15.9)	809 (19.2)	−7.6 (−12.6, −2.7)
Watch television						
No	305 (25.2)	71 (24.3)	168 (22.9)	105 (14.7)	650 (22.0)	−10.5 (−17.0, −3.9)
Yes	288 (21.4)	138 (16.9)	548,919.3)	354 (14.8)	1327 (17.9)	−6.5 (−10.7, −2.5)
**Community-level factors**						
Region of residence						
Tigray	73 (33.1)	28 (31.8)	79 (28.4)	55 (21.9)	235 (28.0)	−11.1 (−19.3, −3.1)
Afar	5 (18.4)	2914.2)	11 (31.2)	8 (23.7)	26 (23.6)	5.3 (−1.5, 12.1)
Amhara	139 (31.3)	32 (18.2)	219 (24.9)	95 (14.5)	485 (22.5)	−16.8 (−26.8, −6.8)
Oromia	163 (20.1)	65 (19.2)	169 (19.8)	130 (16.8)	527 (19.0)	−3.3 (−10.4, 3.9)
Somali	25 (52.2)	9 (22.7)	23 (20.1)	12 (18.1)	70 (25.7)	−34 (−5.3, −15.6)
Benishangul	6 (42.7)	2 (35.6)	8 (22.6)	3 (12.3)	19 (24.4)	−30.3 (− 38.6, −22.0)
SNNPR^b^	44 (17.4)	15 (16.2)	71 (14.9)	25 (7.0)	154 (13.1)	−10.4 (−19.7, − 1.1)
Gambella	4 (31.0)	1 (16.2)	5 (27.2)	5 (28.9)	15 (28.7)	−2.1 (−22.1, 17.9)
Metropolis	134 (18.4)	55 (15.6)	131 (14.9)	125 (13.6)	445 (15.5)	−4.8 (−7.4, −2.1)

n (%): weighted count and proportion for each variable

^a^Diff indicates the point percentage change in prevalence of underweight between 2000 to 2016

^b^*SNNPR* Southern Nations Nationalities and Peoples Region

**Table 2:** Prevalence of overweight/obesity by study variables among urban women in Ethiopia, 2000–2016

Variables	2000	2005	2011	2016	2000–2016	2000–2016
	n (%)	n (%)	n (%)	n (%)	n (%)	^**a**^Diff (95% CI)
**Socioeconomic factors**						
Women’s education						
No schooling	64 (7.4)	30 (11.5)	105 (13.7)	95 (18.5)	294 (12.2)	11.1 (6.1, 16.2)
Primary school	64 (10.3)	30 (10.6)	201 (13.1)	228 (21.7)	523 (15.0)	11.3 (5.1, 17.6)
Secondary and higher	151 (14.2)	98 (17.2)	228 (18.0)	341 (22.2)	818 (18.4)	8.0 (3.1, 12.9)
Women’s employment						
No employment	109 (9.8)	76 (12.0)	206 (13.7)	219 (17.1)	610 (13.5)	7.3 (2.9, 11.6)
Formal employment	133 (14.3)	75 (21.1)	258 (18.0)	391 (27.2)	857 (20.6)	12.8 (7.1, 18.6)
Informal employment	37 (7.4)	6 (5.0)	68 (11.2)	54 (14.3)	165 (10.3)	6.9 (3.5, 13.4)
Marital status						
Not married	88 (8.1)	43 (8.4)	113 (7.4)	143 (11.2)	388 (8.8)	3.1 (−0.4, 6.6)
Currently married	144 (14.4)	85 (21.1)	348 (22.1)	414 (29.4)	992 (22.6)	14.9 (8.3, 21.6)
Formerly married	47 (9.9)	29 (15.5)	72 (15.3)	107 (25.5)	256 (16.5)	15.6 (7.7, 23.9)
Household wealth status						
Poor	135 (8.4)	54 (8.8)	3 (2.8)	3 (2.9)	195 (8.0)	−6.8 (−12.3, −1.3)
Middle	107 (16.6)	54 (21.2)		1 (2.1)	162 (16.6)	−10.3 (−16.3, −4.4)
Rich	8 (11.6)	30 (27.4)	531 (15.4)	661 (22.4)	1229 (18.7)	11.8 (7.5, 16.2)
Toilet facility						
Unimproved	46 (6.2)	50 (9.6)	205 (10.0)	216 (14.2)	517 (10.7)	8.0 (3.3, 12.7)
Improved	233 (12.8)	102 (18.1)	325 (21.7)	437 (28.4)	1096 (20.3)	15.6 (10.6, 20.5)
Source of drinking water						
Unimproved	33 (9.1)	4 (4.3)	37 (12.1)	66 (12.2)	140 (10.8)	3.1 (−4.3, 10.6)
Improved	246 (11.2)	154 (15.0)	496 (15.2)	599 (23.4)	1496 (16.5)	12.1 (7.7, 16.6)
**Demographic factors**						
Women’s age						
15–24 years	85 (6.8)	42 (7.4)	128 (7.4)	131 (10.0)	386 (7.9)	3.2 (−0.1, 6.5)
25–34 years	93 (13.6)	57 (19.9)	197 (18.0)	260 (24.6)	607 (19.4)	11.1 (5.7, 16.4)
35–49 years	101 (16.5)	58 (22.7)	29 (28.1)	274 (37.4)	642 (27.4)	20.9 (11.8, 30.1)
Parity						
None	105 (8.5)	57 (9.7)	145 (8.4)	191 (12.6)	498 (9.8)	4.1 (0.5, 7.7)
1–4 children	115 (13.2)	76 (19.5)	309 (21.2)	408 (31.3)	908 (22.6)	18.1 (12.0, 24.2)
5+ children	59 (13.3)	25 (18.3)	79 (20.5)	65 (23.3)	229 (18.3)	10.0 (−0.2, 20.2)
**Behavioural factors**						
Listening radio						
No	49 (7.8)	33 (15.9)	130 (15.4)	261 (19.3)	474 (15.6)	11.5 (6.1, 16.8)
Yes	230 (12.0)	122 (13.6)	403 (14.9)	403 (23.0)	1158 (15.9)	11.1 (6.8, 15.4)
Read magazine						
No	157 (10.1)	70 (13.2)	282 (14.3)	382 (18.4)	890 (14.6)	8.3 (3.9, 12.6)
Yes	122 (12.1)	88 (15.3)	251 (15.8)	283 (27.4)	743 (17.7)	15.3 (10.5, 20.2)
Watch television						
No	87 (7.2)	29 (9.9)	56 (7.6)	72 (10.1)	244 (8.3)	3.0 (−1.7, 7.6)
Yes	192 (14.3)	129 (15.7)	477 (16.9)	592 (24.8)	1390 (18.8)	10.5 (6.0, 15.1)
**Community-level factors**						
Region of residence						
Tigray	6 (2.7)	4 (4.5)	24 (8.8)	41 (16.1)	75 (9.0)	13.4 (7.3, 19.6)
Afar	4 (12.5)	2 (18.3)	4 (10.1)	6 (19.0)	16 (14.3)	6.5 (−2.8, 15.9)
Amhara	34 (7.5)	20 (11.3)	74 (8.5)	71 (10.7)	198 (9.2)	3.2 (−2.2, 8.6)
Oromia	87 (10.7)	48 (14.2)	121 (14.2)	194 (25.1)	451 (16.2)	14.3 (2.5, 26.1)
Somali	4 (8.3)	11 (26.1)	31 (26.8)	17 (25.0)	62 (23.0)	16.7 (7.7, 25.6)
Benishangul	1 (2.9)	1 (10.8)	3 (9.0)	5 (20.5)	9 (11.3)	17.6 (11.1, 24.1)
SNNPR^b^	27 (10.7)	10 (10.8)	97 (20.5)	60 (16.5)	194 (16.4)	5.8 (−7.8, 19.4)
Gambella	1 (3.8)	1 (5.2)	3 (13.6)	2 (13.5)	6 (10.9)	9.7 (4.2, 15.1)
Metropolis	117 (16.1)	62 (17.6)	177 (20.1)	269 (29.4)	625 (21.7)	13.3 (10.5, 16.0)

n (%): weighted count and proportion for each variable

^a^Diff indicates the point percentage change in prevalence of underweight between 2000 to 2016

^b^SNNPR: Southern Nations Nationalities and Peoples Region

### Trends in underweight and overweight/obesity

**Fig. 1:**
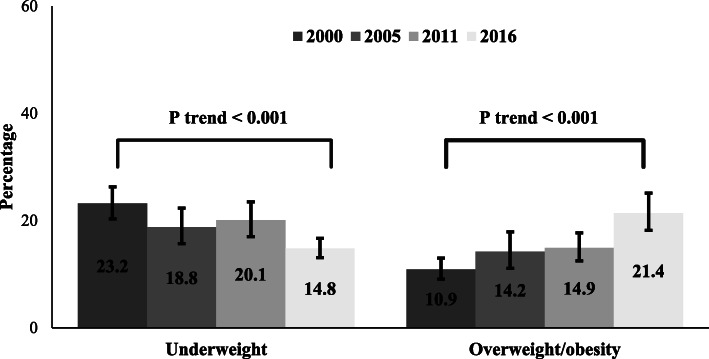
Trends in underweight and overweight/obesity among urban women in Ethiopia from 2000 to 2016. Error bars indicate 95% confidence interval

The proportion of underweight among urban Ethiopian women decreased significantly from 23.2% (95% confidence interval [CI]: 20.3, 26.3%) in 2000 to 14.8% (95% CI: 13.1, 16.7%) in 2016 (Fig. 1). Between 2000 to 2016, the largest decrease in underweight was observed among women who resided in the Benishangul region (Diff = − 30.3; 95% CI: − 38.6, − 22.0), followed by those aged 35–49 years (Diff = − 17.3; 95% CI: − 25.7, − 8.8) (Table 1). The prevalence of overweight/obesity increased significantly from 10.9% (95% CI: 9.1, 13.0%) in 2000 to 21.4% (95% CI: 18.2, 25.1%) in 2016 (Fig. 1). The highest increase in percentage point of overweight/obesity was found among women aged 34–49 years (Diff = 20.9; 95% CI: 11.8, 30.1), followed by those who had 1–4 live birth children (Diff = 18.1; 95% CI: 12.0, 24.2) (Table 2).

### Determinants of underweight among urban Ethiopian women

Over the study period, married women had lower odds of being underweight compared to those who were never married (Adjusted Odds Ratio [AOR] = 0.63; 95% CI: 0.44, 0.91). The odds of women from wealthier households being underweight was significantly lower compared to those who were from poorer households (AOR = 0.69; 95% CI: 0.54, 0.89). Urban women who resided in Oromia (AOR = 0.62; 95% CI: 0.52, 0.86), SNNPR (AOR = 0.43; 95% CI: 0.30, 0.63), and Metropolis (AOR = 0.55; 95% CI: 0.46, 0.67) regions had lower odds of being underweight compared to those who resided in the Tigray region (Table 3).

**Table 3:** Determinants of underweight among urban women in Ethiopia, 2000–2016

Variables	2000	2005	2011	2016	2000–2016	P for trend
	^a^AOR (95% CI)	^a^AOR (95% CI)	^a^AOR (95% CI)	^a^AOR (95% CI)	^a^AOR (95% CI)	
**Socioeconomic factors**						
Women’s education						
No schooling	1.00	1.00	1.00	1.00	1.00	0.005
Primary school	0.86 (0.56, 1.31)	0.97 (0.44, 2.18)	1.27 (0.83, 1.92)	1.00 (0.57, 1.76)	1.00 (0.82, 1.21)	0.115
Secondary and higher	1.10 (0.69, 1.78)	0.93 (0.43, 2.03)	0.99 (0.68, 1.47)	1.35 (0.77, 2.37)	1.28 (0.94, 1.74)	0.225
Women’s employment						
No employment	1.00	1.00	1.00	1.00	1.00	0.265
Formal employment	0.73 (0.49, 1.08)	0.93 (0.64, 1.36)	0.84 (0.64, 1.11)	0.76 (0.56, 1.04)	0.83 (0.70, 0.97)	0.119
Informal employment	0.98 (0.50, 1.92)	1.02 (0.61, 1.70)	0.86 (0.57, 1.27)	0.73 (0.44, 1.22)	0.90 (0.69, 1.17)	0.021
Marital status						
Not married	1.00	1.00	1.00	1.00	1.00	0.330
Currently married	0.73 (0.42, 1.26)	0.38 (0.17, 0.84)	0.59 (0.24, 1.42)	0.82 (0.45, 1.48)	0.63 (0.44, 0.91)	0.001
Formerly married	1.12 (0.69, 1.81)	0.90 (0.45, 1.81)	0.74 (0.32, 1.71)	1.77 (0.91, 3.45)	1.05 (0.76, 1.45)	0.132
Household wealth status						
Poor	1.00	1.00	1.00	1.00	1.00	0.892
Middle	0.77 (0.56, 1.06)	0.89 (0.61, 1.30)	0.10 (0.03, 0.31)	2.99 (0.90, 9.97)	0.76 (0.58, 0.99)	0.931
Rich	0.25 (0.11, 0.55)	0.52 (0.24, 1.11)	0.29 (0.13, 0.68)	0.74 (0.35, 1.56)	0.69 (0.54, 0.89)	0.001
**Demographic factors**						
Women’s age						
15–24 years	1.00	1.00	1.00	1.00	1.00	0.484
25–34 years	0.94 (0.62, 1.41)	1.37 (0.70, 2.66)	1.10 (0.78, 1.55)	0.88 (0.58, 1.34)	0.99 (0.82, 1.20)	0.028
35–49 years	1.63 (0.91, 2.92)	0.81 (0.39, 1.68)	1.29 (0.72, 2.32)	1.00 (0.59, 1.70)	1.21 (0.89, 1.66)	< 0.001
Parity						
None	1.00	1.00	1.00	1.00	1.00	0.460
1–4 children	1.06 (0.69, 1.63)	1.12 (0.46, 2.71)	1.15 (0.53, 2.50)	0.77 (0.46, 1.27)	1.03 (0.74, 1.43)	0.010
5+ children	1.12 (0.55, 2.28)	1.79 (0.54, 5.92)	1.34 (0.41, 4.40)	0.67 (0.27, 1.65)	1.23 (0.76, 1.99)	< 0.001
**Behavioural factors**						
Listening radio						
No	1.00	1.00	1.00	1.00	1.00	0.085
Yes	0.77 (0.67, 1.34)	1.03 (0.61, 1.77)	0.65 (0.49, 0.85)	0.67 (0.45, 0.98)	0.64 (0.66, 0.93)	0.038
Read magazine						
No	1.00	1.00	1.00	1.00	1.00	0.004
Yes	1.28 (0.82, 2.00)	1.04 (0.49, 2.20)	1.06 (0.82, 1.37)	1.23 (0.98, 1.53)	1.14 (0.96, 1.34)	0.116
Watch television						
No	1.00	1.00	1.00	1.00	1.00	0.005
Yes	1.03 (0.79, 1.36)	0.76 (0.50, 1.14)	1.09 (0.82, 1.43)	1.16 (0.61, 2.22)	1.03 (0.84, 1.25)	0.155
**Community-level factor**						
Region of residence						
Tigray	1.00	1.00	1.00	1.00	1.00	0.279
Afar	0.61 (0.41, 0.92)	0.44 (0.17, 1.10)	1.09 (0.68, 1.75)	1.21 (0.78, 1.86)	0.91 (0.67, 1.22)	0.009
Amhara	1.00 (0.53, 1.88)	0.47 90.23, 0.97)	0.81 (0.39, 1.67)	0.54 (0.32, 0.90)	0.75 (0.51, 1.10)	0.014
Oromia	0.70 (0.42, 1.16)	0.57 (0.25, 1.33)	0.70 (0.45, 1.11)	0.82 (0.56, 1.19)	0.67 (0.52, 0.86)	0.969
Somali	3.06 (1.18, 7.89)	0.91 (0.43, 1.93)	0.63 (0.36, 1.11)	0.85 (0.47, 1.53)	1.04 (0.67, 1.63)	0.016
Benishangul	1.63 (1.04, 2.54)	1.20 (0.32, 4.46)	0.54 (0.29, 1.00)	0.49 (0.20, 1.25)	0.87 (0.63, 1.21)	0.003
SNNPR^b^	0.54 (0.23, 1.28)	0.33 (0.16, 0.72)	0.50 (0.27, 0.95)	0.24 (0.14, 0.42)	0.43 (0.30, 0.61)	< 0.001
Gambella	1.17 (0.40, 3.38)	0.93 (0.28, 3.15)	0.72 (0.37, 1.43)	1.32 (0.83, 2.08)	1.10 (0.75, 1.63)	0.521
Metropolis	0.64 (0.42, 0.99)	0.49 (0.28, 0.84)	0.51 (0.34, 0.76)	0.70 (0.54, 0.92)	0.55 (0.46, 0.67)	0.901

^a^AORs of socioeconomic factors were adjusted for demographic, behavioural and community level factors; AORs of demographic factors were adjusted for socioeconomic, behavioural and community level factors; AORs of behavioural factors were adjusted for socioeconomic, demographic, and community level factors; AORs of community-level factors were adjusted for socioeconomic, demographic, and behavioural factors

^b^*SNNPR* Southern Nations Nationalities and Peoples Region

### Determinants of overweight/obesity among urban Ethiopian women

Between 2000 and 2016, urban women who attended secondary or higher education had higher odds of being overweight/obese compared to those who had no schooling (AOR = 1.61; 95% CI: 1.18, 2.21). The likelihood of urban women who were informally employed being overweight/obesity was significantly lower compared to those who were not employed (AOR = 0.69; 95% CI: 0.53; 0.90). Women from wealthier households were more likely to be overweight/obese compared to those who were from poorer households (AOR = 1.64; 95% CI: 1.21, 2.22). Women who listened to the radio had lower odds of being overweight/obese (AOR = 0.76; 95% CI: 0.62, 0.93) compared to those who did not listen to the radio. Women who watched television had higher odds of being overweight/obese (AOR = 2.37; 95% CI: 1.75, 3.21) compared to those who did not watch television. Women who were from Oromia (AOR = 1.95; 95% CI: 1.31, 2.91), Somali (AOR = 4.93; 95% CI: 3.24, 7.49), SNNPR (AOR = 1.89; 95% CI: 1.23, 2.90) and Metropolis (AOR = 2.35; 95% CI: 1.73, 3.21) regions were more likely to be overweight/obese compared to those who resided in the Tigray region (Table 4).

**Table 4:** Determinants of overweight/obesity among urban women in Ethiopia, 2000–2016

Variables	2000	2005	2011	2016	2000–2016	P for trend
	^a^AOR (95% CI)	^a^AOR (95% CI)	^a^AOR (95% CI)	^a^AOR (95% CI)	^a^AOR (95% CI)	
**Socioeconomic factors**						
Women’s education						
No schooling	1.00	1.00	1.00	1.00	1.00	0.126
Primary school	1.65 (1.02, 2.65)	1.64 (0.89, 3.03)	1.24 (0.84, 1.83)	1.19 (0.86, 1.64)	1.33 (1.06,1.68)	0.157
Secondary and higher	2.69 (1.27, 5.71)	1.58 (0.72, 3.48)	1.66 (1.04, 2.67)	1.14 (0.71, 1.84)	1.61 (1.18, 2.21)	0.013
Women’s employment						
No employment	1.00	1.00	1.00	1.00	1.00	0.162
Formal employment	1.26 (0.81, 1.96)	1.31 (0.81, 2.13)	1.02 (0.75, 1.38)	1.42 (1.06, 1.90)	1.23 (1.02, 1.47)	0.044
Informal employment	0.74 (0.39, 1.43)	0.45 (0.17, 1.19)	0.79 (0.50, 1.25)	0.62 (0.43, 0.90)	0.69 (0.53, 0.90)	0.612
Women’s status						
Not married	1.00	1.00	1.00	1.00	1.00	0.182
Currently married	1.51 (0.71, 3.23)	2.11 (0.95, 4.69)	2.03 (1.26, 3.26)	2.07 (1.35, 3.18)	1.87 (1.43, 2.46)	0.057
Formerly married	1.34 (0.54, 3.32)	2.00 (1.03, 3.83)	1.15 (0.51, 2.58)	1.54 (1.05, 2.26)	1.32 (0.94, 1.86)	0.064
Household wealth status						
Poor	1.00	1.00	1.00	1.00	1.00	0.534
Middle	1.43 (0.98, 2.10)	2.97 (1.69, 5.24)	3.12 (1.21, 8.03)	0.94 (0.16, 5.63)	1.46 (1.06, 1.90)	0.008
Rich	0.67 (0.31, 1.48)	3.52 (1.83, 6.77)	3.80 (1.97, 7.33)	4.43 (1.82, 10.79)	1.64 (1.21, 2.22)	0.001
**Demographic factors**						
Women’s age						
15–24 years	1.00	1.00	1.00	1.00	1.00	0.357
25–34 years	1.65 (0.90, 3.00)	2.43 (1.47, 3.99)	2.05 (1.34, 3.16)	2.00 (1.33, 3.02)	2.05 (1.63, 2.59)	0.044
35–49 years	3.50 (1.60, 7.69)	2.53 (1.17, 5.43)	4.64 (2.82, 7.63)	4.35 (2.90, 6.53)	4.47 (3.39, 5.88)	0.115
Parity						
None	1.00	1.00	1.00	1.00	1.00	0.432
1–4 children	0.97 (0.53, 1.77)	0.89 (0.47, 1.67)	1.12 (0.70, 1.78)	1.21 (0.76, 1.94)	1.10 (0.84, 1.44)	0.004
5+ children	0.72 (0.35, 1.47)	1.25 (0.42, 3.77)	0.94 (0.48, 1.87)	0.82 (0.42, 1.61)	0.80 (0.55, 1.17)	0.054
**Behavioural factors**						
Listening radio						
No	1.00	1.00	1.00	1.00	1.00	0.072
Yes	0.88 (0.48, 1.62)	0.63 (0.30, 1.35)	0.70 (0.47, 1.04)	0.69 (0.54, 0.88)	0.76 (0.62, 0.93)	0.009
Read magazine						
No	1.00	1.00	1.00	1.00	1.00	0.541
Yes	0.67 (0.42, 1.08)	1.12 (0.64, 1.95)	1.04 (0.78, 1.39)	1.56 (1.14, 2.14)	1.12 (0.93, 1.35)	< 0.001
Watch television						
No	1.00	1.00	1.00	1.00	1.00	0.959
Yes	1.66 (0.96, 2.87)	1.43 (0.68, 3.03)	2.45 (1.30, 4.60)	2.50 (1.41, 4.45)	2.37 (1.75, 3.21)	0.002
**Community-level factors**						
Region of residence						
Tigray	1.00	1.00	1.00	1.00	1.00	< 0.001
Afar	4.54 (2.25, 9.15)	3.66 (0.89, 15.01)	1.08 (0.48, 2.45)	1.34 (0.65, 2.78)	1.71 (1.13, 2.60)	0.760
Amhara	1.67 (1.15, 6.18)	3.04 (0.83, 11.19)	0.98 (0.42, 2.28)	0.62 (0.35, 1.11)	1.02 (0.68, 1.52)	0.176
Oromia	4.59 (2.04, 10.33)	3.53 (0.99, 12.45)	1.58 (0.76, 3.27)	1.62 (0.85, 3.10)	1.95 (1.31, 2.91)	0.327
Somali	6.66 (1.48, 29.4)	6.20 (2.41, 16.00)	5.81 (2.70, 12.52)	3.13 (1.86, 5.26)	4.93 (3.24, 7.49)	0.875
Benishangul	1.34 (0.20, 8.91)	4.78 (0.98, 23.24)	1.08 (0.46, 2.52)	1.85 (0.98, 3.50)	1.59 (1.01, 2.48)	0.083
SNNPR	3.87 (0.94, 15.97)	2.44 (0.78, 7.58)	2.39 (1.17, 4.87)	1.29 (0.73, 2.29)	1.89 (1.23, 2.90)	0.802
Gambella	1.64 (0.64, 4.18)	0.88 (0.26, 2.93)	1.43 (0.56, 3.63)	0.99 (0.56, 1.78)	1.21 (0.76, 1.87)	0.063
Metropolis	4.20 (2.03, 8.68)	2.45 (0.99, 6.04)	2.15 (1.16, 3.99)	2.14 (1.40, 3.26)	2.35 (1.73, 3.21)	< 0.001

^a^AORs of socioeconomic factors were adjusted for demographic, behavioural and community level factors; AORs of demographic factors were adjusted for socioeconomic, behavioural and community level factors; AORs of behavioural factors were adjusted for socioeconomic, demographic, and community level factors; AORs of community-level factors were adjusted for socioeconomic, demographic, and behavioural factors

^b^*SNNPR* Southern Nations Nationalities and Peoples Region

## Discussion

The prevalence of underweight in urban Ethiopian women decreased from 23.2% in 2000 to 14.8% in 2016, while overweight/obesity prevalence increased from 10.9% in 2000 to 21.4% in 2016. Factors associated with a lower likelihood of women being underweight in Urban Ethiopia included higher household wealth, never being married, and residence in Oromia, SNNPR, and Metropolis regions. Belonging to wealthier households, higher educational attainment and watching TV were associated with urban Ethiopian women being overweight/obese. Informal employment and listening to the radio were associated with a reduced likelihood of women being overweight/obese.

Evidence has shown that the relationship between household wealth and underweight and/or overweight/obesity differs across socioeconomic levels at the global, regional, national and subnational levels [22, 41, 46–49]. In LMICs, individuals from wealthy households have a higher risk of being overweight/obese compared to those from poorer households [46–49]. In the present study, women from wealthier households were more likely to be overweight/obese but less likely to be underweight compared to those who were from poorer households. These findings are similar to evidence from South Asian [18, 50] and sub-Saharan Africa countries [23, 39], which showed that wealthier women were more likely to be overweight/obese but less likely to be underweight compared to counterparts. A possible explanation for the high likelihood of urban Ethiopian women from wealthier households being overweight/obese may be due to lifestyle and dietary choices. Women from wealthier households may be less physically active and also have better healthy dietary choices (such as poor consumption of fruits and vegetables, and a higher intake of highly caloric foods) compared to those who reside in poor households [51, 52]. Our study also showed that women who were from wealthier households were less likely to be underweight compared to those from poorer households. The economic disadvantage of urban women from poorer households may explain the negative relationship between higher households’ wealth and underweight [53]. Our findings suggest that interventions to reduce overweight/obesity and NCDs and improve underweight should target women from both poor and rich households in urban Ethiopia.

Studies from high-income countries have shown that women who attained higher education had reduced risk of developing overweight/obesity compared to those with lower education [54–56]. However, in LMICs, educated women were more likely to be overweight/obese compared to those with no education [56, 57]. Our study indicated that urban women who had secondary or higher education were more likely to be overweight/obese compared to those who had no schooling. The positive association between higher educational attainment and overweight/obesity among women has also been reported in studies conducted in Ghana [39], Bangladesh [19], regional and national levels in Ethiopia [22, 23]. This relationship may be due to a range of factors. First, women in LMICs perceive overweight/obesity or ‘round body’ frame as an indicator of socioeconomic success, and this perception possibly allows women to ‘celebrate’ increasing weight gain [39, 58]. Second, it may be due to a shift from more physically active occupations (e.g. construction labour works) to less active or sedentary occupations (e.g. office works) [23, 59].

Consistent with previously published studies [60–62], the current study found that urban Ethiopian women who were employed in manual jobs had a lower risk of being overweight/obese compared to those who were not in employment. There are two likely explanations for the observed relationship between informal employment and overweight/obesity. Firstly, informally employed women are often employed in labour-intensive or physically active jobs like construction labourer, and this may be associated with negative energy balance [62]. Secondly, the limited purchasing power of women, due to lower wages from informal employment, may not allow women to purchase energy-dense or junk foods [63]. These findings suggest that health and social policy interventions for urban Ethiopian women should focus on modifiable socio-economic factors such as improvement in female education and employment opportunities to reduce the burden of overweight/obesity. Additionally, health education on physical activity and healthy dietary options are also essential given the improvement in socio-economics status of women in LMICs which may be associated with overweight/obesity among women in these settings [64, 65].

Sedentary behaviours (including watching television) and inadequate physical activity have been documented as risk factors for overweight/obesity [66, 67]. Consistent with this evidence, the present study showed that urban women who watched television had higher odds of being overweight/obese compared to those who did not watch television. Studies conducted in Ghana [39], Bangladesh [66], and Myanmar [68] have also reported the association between watching television and overweight/obesity. This finding may be related to a reduced level of physical activity among individuals as a result of increased sitting time [66, 67]. In addition, in LMICs, having a television can also be a proxy indicator for the higher socioeconomic status of women, which may increase the risk of exposure to energy-dense and junk foods [51, 52]. Urban women who listened to the radio were less likely to be overweight/obese compared to that not-listened radio. It is possible that urban Ethiopian women who resided in poorer households or urban slums were more likely to listen to the radio compared to those who were from wealthy households. Past studies have suggested that health promotion through electronic media (such as radio and television) showed improvement in the awareness of dietary habits and active lifestyle of women [69, 70]. The use of electronic media for improving physical activity and healthy dietary choices of urban Ethiopian women is warranted.

This study has limitations. First, this study used cross-sectional data which presents difficulty in establishing a temporal association between the study factors and the outcome measures. Nevertheless, the observed associations are consistent with cohort [71] and cross-sectional studies from LMICs [23, 39, 66, 68]. Second, the study was limited by the non-availability of data on key confounders such as dietary intake, length of time in watching TV, physical activity and total energy expenditure of the urban women, as the EDHS did not collect information on these variables. Third, the study factors were measured based on self-report questionnaires is a source of measurement bias which may either over- or under-estimate the measure of association between the study factors and outcome variables. Despite the above limitations, the present study provides nationally representative data on underweight and overweight/obesity in Ethiopia. The use of a standardized questionnaire is a strength of the current study as it improves the internal validity, as well as the accuracy of the estimated measure of association.

## Conclusion

The present study shows that the prevalence of underweight among urban Ethiopian women improved from 23.2% in 2000 to 14.8% in 2016, while overweight/obesity prevalence increased from 10.9 to 21.4% over the same period. Key modifiable factors negatively associated with underweight included women who resided in wealthy households and never married, while the factors associated with overweight/obesity were residence in rich households and higher education attainment. Women who were informally employed and listened to the radio were less likely to be overweight/obese. Locally-relevant policy and interventions should not only target improvement in the socioeconomic status of Ethiopian women but should also focus on the education of women around the benefits of regular physical activity and healthy dietary choices.

## Supplementary information

**Additional file 1.** Characteristics of urban women in Ethiopia, 2000–2016. n (%): weighted count and proportions for each variable. *SNNPR: Southern Nations Nationalities and Peoples Region.

**Additional file 2.** Percentage point change in the prevalence of underweight by study factors, 2000–2016. n (%): weighted count and proportion for each variable. *Diff indicates the point percentage change in prevalence of underweight between 2000 to 2016.

**Additional file 3.** Percentage point change in the prevalence of overweight/obesity by study factors, 2000–2016. n (%): weighted count and proportion for each variable. *Diff indicates the point percentage change in prevalence of underweight between 2000 to 2016.

**Additional file 4.** Bar graphs showing trends of underweight and overweight/obesity by each study variables from 2000 to 2016.

## Data Availability

The analysis was based on the datasets collected as the Ethiopian Demographic Health Survey. Information on the data and content can be accessed at https://dhsprogram.com/data/available-datasets.cfm.

## Abbreviations

BMI: Body mass index
CI: Confidence interval
CSA: Central Statistics Agency
DHS: Demographic and Health Survey
EA: Enumeration Areas
EDHS: Ethiopian Demographic and Health Survey
ICF: Inner City Fund
LMICs: Lower and Middle income Countries
NCD: Non-communicable diseases
NRERC: National Research Ethics Review Committee
OR: Odds ratio
SDG: Sustainable Development Goals
SNNPR: Southern Nations Nationalities and Peoples Regions
USAID: United States Agency for International Development
WHO: World Health Organization

## References

[1] Batal M, Steinhouse L, Delisle H (2018). The nutrition transition and the double burden of malnutrition. Med Sante Trop.

[2] Broyles ST, Denstel KD, Church TS, Chaput JP, Fogelholm M, Hu G, Kuriyan R, Kurpad A, Lambert EV, Maher C (2015). The epidemiological transition and the global childhood obesity epidemic. Int J Obes Suppl.

[3] Shetty P (2013). Nutrition transition and its health outcomes. Indian J Pediatr.

[4] World Health Organisation. The double burden of malnutrition: policy brief. Geneva: World Health Organisation; 2016.

[5] Amugsi DA, Dimbuene ZT, Mberu B, Muthuri S, Ezeh AC (2017). Prevalence and time trends in overweight and obesity among urban women: an analysis of demographic and health surveys data from 24 African countries, 1991-2014. BMJ Open.

[6] Lash MM, Armstrong A (2009). Impact of obesity on women's health. Fertil Steril.

[7] Must A, Naumova EN, Phillips SM, Blum M, Dawson-Hughes B, Rand WM (2005). Childhood overweight and maturational timing in the development of adult overweight and fatness: the Newton girls study and its follow-up. Pediatrics.

[8] Himes JH (2006). Examining the evidence for recent secular changes in the timing of puberty in US children in light of increases in the prevalence of obesity. Mol Cell Endocrinol.

[9] Freire WB, Waters WF, Rivas-Marino G, Belmont P (2018). The double burden of chronic malnutrition and overweight and obesity in Ecuadorian mothers and children, 1986-2012. Nutr Health.

[10] Whitaker RC (2004). Predicting preschooler obesity at birth: the role of maternal obesity in early pregnancy. Pediatrics.

[11] Bygbjerg IC (2012). Double burden of noncommunicable and infectious diseases in developing countries. Science.

[12] Allen LN, Pullar J, Wickramasinghe KK, Williams J, Roberts N, Mikkelsen B, Varghese C, Townsend N (2018). Evaluation of research on interventions aligned to WHO ‘best buys’ for NCDs in low-income and lower-middle-income countries: a systematic review from 1990 to 2015. BMJ Glob Health.

[13] NCD Risk Factor Collaboration (NCD-RisC) (2016). Trends in adult body-mass index in 200 countries from 1975 to 2014: a pooled analysis of 1698 population-based measurement studies with 19.2 million participants. Lancet.

[14] Afshin A, Forouzanfar MH, Reitsma MB, Sur P, Estep K, Lee A, Marczak L, Mokdad AH, Moradi-Lakeh M, Naghavi M (2017). Health effects of overweight and obesity in 195 countries over 25 years. N Engl J Med.

[15] Overweight and obesity In: https://www.who.int/news-room/fact-sheets/detail/obesity-and-overweight. [Accessed 15 Aug 2019].

[16] Prevalence of underweight among adults, BMI < 18, crude estimates by WHO region [http://apps.who.int/gho/data/view.main.NCDBMILT18CREGv].

[17] Rawal LB, Kanda K, Mahumud RA, Joshi D, Mehata S, Shrestha N, Poudel P, Karki S, Renzaho A (2018). Prevalence of underweight, overweight and obesity and their associated risk factors in Nepalese adults: data from a nationwide survey, 2016. PLoS One.

[18] Biswas T, Garnett SP, Pervin S, Rawal LB (2017). The prevalence of underweight, overweight and obesity in Bangladeshi adults: data from a national survey. PLoS One.

[19] Kamal SM, Hassan CH, Alam GM (2015). Dual burden of underweight and overweight among women in Bangladesh: patterns, prevalence, and sociodemographic correlates. J Health Popul Nutr.

[20] Ajayi IO, Adebamowo C, Adami H-O, Dalal S, Diamond MB, Bajunirwe F, Guwatudde D, Njelekela M, Nankya-Mutyoba J, Chiwanga FS (2016). Urban–rural and geographic differences in overweight and obesity in four sub-Saharan African adult populations: a multi-country cross-sectional study. BMC Public Health.

[21] Seyda Seydel G, Kucukoglu O, Altinbasv A, Demir OO, Yilmaz S, Akkiz H, Otan E, Sowa JP, Canbay A (2016). Economic growth leads to increase of obesity and associated hepatocellular carcinoma in developing countries. Ann Hepatol.

[22] Tebekaw Y, Teller C, Colón-Ramos U (2014). The burden of underweight and overweight among women in Addis Ababa, Ethiopia. BMC Public Health.

[23] Abrha S, Shiferaw S, Ahmed KY (2016). Overweight and obesity and its socio-demographic correlates among urban Ethiopian women: evidence from the 2011 EDHS. BMC Public Health.

[24] Rothman KJ, Lash TL, Greenland S (2008). Modern epidemiology.

[25] Ethiopia sensitizes stakeholders on its new food and nutrition policy [https://www.unicef.org/ethiopia/stories/ethiopia-sensitizes-stakeholders-its-new-food-and-nutrition-policy].

[26] Ethiopia approves national food and nutrition policy [https://ethiopia.savethechildren.net/news/ethiopia-approves-national-food-and-nutrition-policy].

[27] SDG-UN (2015). Transforming our world: the 2030 agenda for sustainable development.

[28] World Health Organisation. Global action plan for the prevention and control of noncommunicable diseases: 2013–2020. Geneva: World Health Organisation; 2013.

[29] Croft TN, Marshall AMJ, Allen CK (2018). Guide to demographic and health survey statistics.

[30] Central Statistics Agency (CSA) [Ethiopia] and ORC Macro (2001). Ethiopian demographic and health survey 2000.

[31] Central Statistics Agency (CSA) [Ethiopia] and ORC Macro (2006). Ethiopia demographic and health survey 2005.

[32] Central Statistics Agency (CSA) [Ethiopia] and ICF International (2012). Ethiopia demographic and health survey 2011.

[33] Central Statistics Agency (CSA) [Ethiopia] and ICF International (2016). Ethiopia demographic and health survey 2016.

[34] Ogbo FA, Nguyen H, Naz S, Agho KE, Page A (2018). The association between infant and young child feeding practices and diarrhoea in Tanzanian children. Trop Med Health.

[35] Ogbo FA, Page A, Agho KE, Claudio F (2015). Determinants of trends in breast-feeding indicators in Nigeria, 1999-2013. Public Health Nutr.

[36] Ahmed KY, Page A, Arora A, Ogbo FA (2019). Trends and determinants of early initiation of breastfeeding and exclusive breastfeeding in Ethiopia from 2000 to 2016. Int Breastfeed J.

[37] Tanwi TS, Chakrabarty S, Hasanuzzaman S (2019). Double burden of malnutrition among ever-married women in Bangladesh: a pooled analysis. BMC Womens Health.

[38] Araujo FG, Velasquez-Melendez G, Felisbino-Mendes MS. Prevalence trends of overweight, obesity, diabetes and hypertension among Brazilian women of reproductive age based on sociodemographic characteristics. Health Care Women Int. 2019;40(4):386–406. 10.1080/07399332.2019.1570516 30986134

[39] Doku DT, Neupane S (2015). Double burden of malnutrition: increasing overweight and obesity and stall underweight trends among Ghanaian women. BMC Public Health.

[40] Hasan M, Sutradhar I, Shahabuddin A, Sarker M (2017). Double burden of malnutrition among Bangladeshi women: a literature review. Cureus.

[41] Neupane S, Prakash KC, Doku DT (2016). Overweight and obesity among women: analysis of demographic and health survey data from 32 sub-Saharan African countries. BMC Public Health.

[42] Abou-ElWafa HS, El-Gilany AH. Maternal work and exclusive breastfeeding in Mansoura, Egypt. Fam Pract. 2018;36(5):568–72. 10.1093/fampra/cmy120 30508085

[43] Filmer D, Pritchett LH (2001). Estimating wealth effects without expenditure data--or tears: an application to educational enrollments in states of India. Demography.

[44] Lakew Y, Tabar L, Haile D (2015). Socio-medical determinants of timely breastfeeding initiation in Ethiopia: evidence from the 2011 nationwide demographic and health survey. Int Breastfeed J.

[45] Ogbo FA, Page A, Idoko J, Claudio F, Agho KE (2015). Trends in complementary feeding indicators in Nigeria, 2003–2013. BMJ Open.

[46] Christensen DL, Eis J, Hansen AW, Larsson MW, Mwaniki DL, Kilonzo B, Tetens I, Boit MK, Kaduka L, Borch-Johnsen K (2008). Obesity and regional fat distribution in Kenyan populations: impact of ethnicity and urbanization. Ann Hum Biol.

[47] Fezeu L, Minkoulou E, Balkau B, Kengne AP, Awah P, Unwin N, Alberti GK, Mbanya JC (2006). Association between socioeconomic status and adiposity in urban Cameroon. Int J Epidemiol.

[48] Lopez RP (2007). Neighborhood risk factors for obesity. Obesity (Silver Spring, Md).

[49] Sundquist K, Malmstrom M, Johansson SE (2004). Neighbourhood deprivation and incidence of coronary heart disease: a multilevel study of 2.6 million women and men in Sweden. J Epidemiol Community Health.

[50] Bishwajit G (2017). Household wealth status and overweight and obesity among adult women in Bangladesh and Nepal. Obes Sci Pract.

[51] McLaren L (2007). Socioeconomic status and obesity. Epidemiol Rev.

[52] Fernald LC (2007). Socio-economic status and body mass index in low-income Mexican adults. Soc Sci Med.

[53] Bhurosy T, Jeewon R (2014). Overweight and obesity epidemic in developing countries: a problem with diet, physical activity, or socioeconomic status?. ScientificWorldJournal.

[54] Ogden CL, Fakhouri TH, Carroll MD, Hales CM, Fryar CD, Li X, Freedman DS (2017). Prevalence of obesity among adults, by household income and education - United States, 2011-2014. MMWR Morb Mortal Wkly Rep.

[55] Murakami K, Ohkubo T, Hashimoto H (2017). Distinct association between educational attainment and overweight/obesity in unmarried and married women: evidence from a population-based study in Japan. BMC Public Health.

[56] Cohen AK, Rai M, Rehkopf DH, Abrams B (2013). Educational attainment and obesity: a systematic review. Obes Rev.

[57] Ford ND, Patel SA, Narayan KM (2017). Obesity in low- and middle-income countries: burden, drivers, and emerging challenges. Annu Rev Public Health.

[58] Martorell R, Khan LK, Hughes ML, Grummer-Strawn LM (2000). Obesity in women from developing countries. Eur J Clin Nutr.

[59] Ziraba AK, Fotso JC, Ochako R (2009). Overweight and obesity in urban Africa: a problem of the rich or the poor?. BMC Public Health.

[60] Goryakin Y, Suhrcke M (2014). Economic development, urbanization, technological change and overweight: what do we learn from 244 demographic and health surveys?. Econ Hum Biol.

[61] Oddo VM, Bleich SN, Pollack KM, Surkan PJ, Mueller NT, Jones-Smith JC (2017). The weight of work: the association between maternal employment and overweight in low- and middle-income countries. Int J Behav Nutr Phys Act.

[62] Oddo VM, Surkan PJ, Hurley KM, Lowery C, de Ponce S, Jones-Smith JC (2018). Pathways of the association between maternal employment and weight status among women and children: qualitative findings from Guatemala. Matern Child Nutr.

[63] Günther I, Launov A (2012). Informal employment in developing countries: opportunity or last resort?. J Dev Econ.

[64] Frantz JM, Ngambare R (2013). Physical activity and health promotion strategies among physiotherapists in Rwanda. Afr Health Sci.

[65] Patnode CD, Evans CV, Senger CA, Redmond N, Lin JS (2017). U.S. preventive services task force evidence syntheses, formerly systematic evidence reviews. Behavioral counseling to promote a healthful diet and physical activity for cardiovascular disease prevention in adults without known cardiovascular disease risk factors: updated systematic review for the us preventive services task Force.

[66] Ghose B (2017). Frequency of TV viewing and prevalence of overweight and obesity among adult women in Bangladesh: a cross-sectional study. BMJ Open.

[67] Healy GN, Wijndaele K, Dunstan DW, Shaw JE, Salmon J, Zimmet PZ, Owen N (2008). Objectively measured sedentary time, physical activity, and metabolic risk: the Australian diabetes, obesity and lifestyle study (AusDiab). Diabetes Care.

[68] Das Gupta R, Sajal IH, Hasan M, Sutradhar I, Haider MR, Sarker M (2019). Frequency of television viewing and association with overweight and obesity among women of the reproductive age group in Myanmar: results from a nationwide cross-sectional survey. BMJ Open.

[69] Miles A, Rapoport L, Wardle J, Afuape T, Duman M (2001). Using the mass-media to target obesity: an analysis of the characteristics and reported behaviour change of participants in the BBC’s ‘fighting fat, fighting fit’ campaign. Health Educ Res.

[70] Wardle J, Rapoport L, Miles A, Afuape T, Duman M (2001). Mass education for obesity prevention: the penetration of the BBC’s ‘fighting fat, fighting fit’ campaign. Health Educ Res.

[71] Rai RK, Jaacks LM, Bromage S, Barik A, Fawzi WW, Chowdhury A (2018). Prospective cohort study of overweight and obesity among rural Indian adults: sociodemographic predictors of prevalence, incidence and remission. BMJ Open.

