# Supplementary material for: Trends and determinants of underweight and overweight/obesity among urban Ethiopian women from 2000 to 2016
Source: BMC Public Health. 2020 Aug 24;20:1276. doi: 10.1186/s12889-020-09345-6 (PMC7447570; doi:10.1186/s12889-020-09345-6)
Supplement: Supplementary file 1 — Additional file 1. Characteristics of urban women in Ethiopia, 2000–2016. n (%): weighted count and proportions for each variable. *SNNPR: Southern Nations Nationalities and Peoples Region. [file 12889_2020_9345_MOESM1_ESM.docx]

**Additional file 1**

Characteristics of urban women in Ethiopia, 2000–2016

| **Variables** | **2000** | **2005** | **2011** | **2016** | **2000–2016** |
| --- | --- | --- | --- | --- | --- |
|  | **n (%)** | **n (%)** | **n (%)** | **n (%)** | **n (%)** |
| **Socioeconomic factors** |  |  |  |  |  |
| Women’s education |  |  |  |  |  |
| No schooling | 871 (34.1) | 259 (23.3) | 767 (21.5) | 516 (16.6) | 2413 (23.3) |
| Primary school | 624 (24.4) | 282 (25.4) | 1533 (43.0) | 1052 (33.9) | 3491 (33.7) |
| Secondary and higher | 1064 (41.6) | 571 (51.4) | 1269 (35.6) | 1539 (49.6) | 4442 (42.9) |
| Women’s employment |  |  |  |  |  |
| No employment | 1115 (44.0) | 635 (57.2) | 1501 (42.3) | 1285 (41.4) | 4537 (44.1) |
| Formal employment | 928 (36.6) | 356 (32.0) | 1437 (40.5) | 1440 (46.4) | 4161 (40.4) |
| Informal employment | 494 (19.5) | 120 (10.8) | 608 (17.1) | 381 (12.3) | 1602 (15.6) |
| Marital status |  |  |  |  |  |
| Not married | 1086 (42.5) | 519 (46.7) | 1525 (42.7) | 1275 (41.1) | 4406 (42.6) |
| Currently married | 999 (39.0) | 404 (36.3) | 1572 (44.1) | 1411 (45.4) | 4386 (43.4) |
| Formerly married | 474 (18.5) | 189 (17.0) | 472 (13.2) | 420 (13.5) | 1554 (15.0) |
| Household wealth status |  |  |  |  |  |
| Poor | 572 (23.5) | 14 (1.3) | 94 (2.6) | 114 (3.7) | 795 (7.8) |
| Middle | 437 (18.0) | 8 (1.0) | 37 (1.0) | 41 (1.3) | 523 (5.1) |
| Rich | 1421 (58.5) | 1090 (97.7) | 3438 (96.4) | 2951 (95.0) | 8900 (87.1) |
| Toilet system |  |  |  |  |  |
| Unimproved | 740 (18.9) | 520 (48.1) | 2060 (58.0) | 1518 (49.7) | 4839 (47.2) |
| Improved | 1818 (71.1) | 562 (51.9) | 1494 (42.0) | 1538 (50.3) | 5412 (52.8) |
| Source of drinking water |  |  |  |  |  |
| Unimproved | 363 (14.2) | 83 (7.5) | 306 (8.6) | 542 (17.5) | 1294 (12.5) |
| Improved | 2196 (85.8) | 1029 (92.5) | 3269 (91.4) | 2564 (82.6) | 9052 (87.5) |
| **Demographic factors** |  |  |  |  |  |
| Women’s age |  |  |  |  |  |
| 15-24 years | 1257 (49.1) | 567 (51.0) | 1733 (48.6) | 1317 (42.4) | 4875 (47.1) |
| 25-34 years | 687 (26.9) | 288 (25.9) | 1094 (30.7) | 1057 (34.0) | 3125 (30.2) |
| 35-49 years | 614 (24.0) | 257 (23.1) | 742 (20.8) | 732 (23.6) | 2345 (22.7) |
| Parity |  |  |  |  |  |
| None | 1237 (48.4) | 585 (52.6) | 1728 (48.4) | 1520 (48.9) | 5070 (49.0) |
| 1-4 children | 878 (34.3) | 388 (34.9) | 1457 (40.8) | 1305 (42.0) | 4028 (38.9) |
| 5+ children | 444 (17.3) | 139 (12.5) | 384 (10.8) | 281 (9.1) | 1248 (12.1) |
| **Behavioural factors** |  |  |  |  |  |
| Listening radio |  |  |  |  |  |
| No | 629 (24.6) | 211 (19.0) | 849 (23.8) | 1355 (43.6) | 3044 (29.5) |
| Yes | 1923 (75.4) | 899 (81.0) | 2714 (76.2) | 1751 (56.4) | 7287 (70.5) |
| Read magazine |  |  |  |  |  |
| No | 1543 (60.3) | 529 (47.8) | 1971 (55.4) | 2074 (66.8) | 6116 (59.2) |
| Yes | 1015 (39.7) | 577 (52.2) | 1587 (44.6) | 1032 (33.2) | 4211 (40.8) |
| Watch television |  |  |  |  |  |
| No | 1211 (47.3) | 293 (26.3) | 735 (20.6) | 714 (23.0) | 2953 (28.6) |
| Yes | 1347 (52.7) | 819 (73.7) | 2833 (79.4) | 2392 (77.0) | 7391 (71.5) |
| **Community-level factors** |  |  |  |  |  |
| Region of residence |  |  |  |  |  |
| Tigray | 220 (8.6) | 89 (8.0) | 278 (7.8) | 253 (8.2) | 840 (8.1) |
| Afar | 29 (1.1) | 13 (1.1) | 36 (1.0) | 33 (1.1) | 110 (1.1) |
| Amhara | 445 (17.4) | 176 (15.9) | 878 (24.6) | 659 (21.2) | 2158 (20.9) |
| Oromia | 810 (31.7) | 340 (30.6) | 855 (24.0) | 774 (24.9) | 2780 (26.9) |
| Somali | 48 (1.9) | 40 (3.6) | 115 (3.2) | 68 (2.2) | 271 (2.6) |
| Benishangul | 15 (0.6) | 6 (0.5) | 36 (1.0) | 23 (0.7) | 79 (0.8) |
| SNNPR* | 252 (9.9) | 90 (8.0) | 473 (13.3) | 364 (11.7) | 1180 (11.4) |
| Gambella | 11 (0.4) | 3 (0.3) | 19 (5.4) | 18 (0.6) | 51 (0.5) |
| Metropolis | 728 (28.5) | 355 (32.0) | 880 (24.6) | 914 (29.4) | 2877 (27.8) |

**n (%): weighted count and proportions for each variable**

***SNNPR: Southern Nations Nationalities and Peoples Region**
