# Supplementary material for: Trends and determinants of underweight and overweight/obesity among urban Ethiopian women from 2000 to 2016
Source: BMC Public Health. 2020 Aug 24;20:1276. doi: 10.1186/s12889-020-09345-6 (PMC7447570; doi:10.1186/s12889-020-09345-6)
Supplement: Supplementary file 2 — Additional file 2. Percentage point change in the prevalence of underweight by study factors, 2000–2016. n (%): weighted count and proportion for each variable. *Diff indicates the point percentage change in prevalence of underweight between 2000 to 2016. [file 12889_2020_9345_MOESM2_ESM.docx]

**Additional file 2**

**Percentage point change in the prevalence of underweight by study factors, 2000–2016**

| **Variables** | **2000** | **2000–2005** | **2005** | **2005–2011** | **2011** | **2011–2016** | **2016** | **2000–2016** |
| --- | --- | --- | --- | --- | --- | --- | --- | --- |
|  | **n (%)** | ***Diff (95% CI)** | **n (%)** | ***Diff (95% CI)** | **n (%)** | ***Diff (95% CI)** | **n (%)** | ***Diff (95% CI)** |
| **Socioeconomic factors** |  |  |  |  |  |  |  |  |
| Women’s education |  |  |  |  |  |  |  |  |
| No schooling | 230 (26.4) | -5.0 (-13.4, 3.4) | 55 (21.4) | -1.1 (-9.1, 6.9) | 155 (20.3) | -7.3 (-13.5, -1.2) | 67 (12.9) | -13.5 (-20.1, -6.8) |
| Primary school | 121 (19.3) | 0.2 (-7.0, 7.3) | 55 (19.5) | 2.9 (-4.4, 10.2) | 344 (22.4) | -9.7 (-15.0, -4.4) | 134 (12.7) | -6.6 (-11.7, -1.5) |
| Secondary and higher | 242 (22.8) | -5.5 (-11.5, 0.5) | 99 (17.3) | -0.2 (-5.7, 5.3) | 217 (17.1) | -0.3 (-4.6, 0.4) | 259 (16.8) | -6.0 (-10.8, -1.1) |
| Women’s employment |  |  |  |  |  |  |  |  |
| No employment | 278 (25.0) | -5.6 (-11.8, 0.6) | 123 (19.3) | 1.9 (-3.6, 7.4) | 318 (21.2) | -3.6 (-8.6, 1.3) | 226 (17.6) | -7.4 (-13.1, -1.6) |
| Formal employment | 176 (19.0) | -2.6 (-9.1, 3.8) | 58 (16.4) | 1.7 (-4.3, 7.8) | 260 (18.1) | -5.4 (-10.0, -0.8) | 183 (12.7) | -6.3 (-11.5, -1.1) |
| Informal employment | 129 (26.2) | -2.7 (-12.7, 7.2) | 28 (23.5) | -2.0 (-10.0, 6.1) | 131 (21.5) | -8.3 (-15.6, -0.9) | 50 (13.2) | -13.0 (-22.3, -3.6) |
| Marital status |  |  |  |  |  |  |  |  |
| Not married | 256 (23.6) | -1.8 (-8.0, 4.4) | 113 (21.8) | 1.4 (-4.7, 7.5) | 354 (23.2) | -4.0 (-8.7, 0.8) | 246 (19.3) | -4.4 (-9.1, 0.5) |
| Currently married | 204 (20.5) | -7.3 (-12.2, -2.4) | 53 (13.1) | 3.4 (-2.6, 9.4) | 260 (16.5) | -7.2 (-12.7, -1.6) | 132 (9.4) | -11.1 (-15.3, -6.8) |
| Formerly married | 132 (27.9) | -5.3 (-16.2, 5.6) | 43 (22.6) | -1.1 (-11.6, 9.4) | 101 (21.5) | -2.2 (-9.5, 5.1) | 81 919.3) | -8.7 (-16.5, -0.6) |
| Household wealth status |  |  |  |  |  |  |  |  |
| Poor | 427 (26.5) | 4.1 (-13.2, 17.9) | 138 (22.4) | 28.3 (0.1, 49.7) | 48 (51.0) | -35.4 (6.1, -16.3) | 14 (12.5) | -11.3 (-22.4, -0.2) |
| Middle | 121 (18.8) | -2.4 (-2.9, 79.9) | 42 (16.4) | ---5.7 (-88.1, -4.3) | 4 (10.7) | 24.1 (-2.4, 50.5) | 14 (34.8) | 16.3 (-9.3, 42.0) |
| Rich | 4 (6.3) | 3.8 (-2.4, 5.1) | 11 (10.1) | 9.2 (-9.3, 41.9) | 716 (19.3) | -4.7 (-6.7, 5.3) | 431 (14.6) | -9.9 (-13.3, -6.5) |
| Toilet facility |  |  |  |  |  |  |  |  |
| Unimproved | 196 (26.4) | -4.4 (-11.9, 3.2) | 115 (22.1) | 1.1 (-5.3, 7.5) | 477 (23.2) | -8.3 (-13.3, -3.3) | 226 (14.9) | -11.6 (-18.0, -5.2) |
| Improved | 397 (21.8) | -6.4 (-11.5, -1.4) | 87 (15.4) | 0.3 (-4.0, 4.7) | 235 (15.8) | -1.0 (-4.9, 3.0) | 227 (14.8) | -7.0 (-11.8, -2.3) |
| Source of drinking water |  |  |  |  |  |  |  |  |
| Unimproved | 89 (24.5) | -3.4 (-13.1, 6.3) | 18 (21.1) | 0.1 (-10.2, 10.5) | 65 (21.3) | -5.5 (-13.0, 2.0) | 85 (15.8) | -8.7 (-15.3, -2.1) |
| Improved | 504 (23.00 | -4.3 (-9.2, 0.5) | 192 (18.6) | 1.3 (-3.4, 6.1) | 651 (19.9) | -5.4 (-9.3, -1.4) | 374 (14.6) | -8.4 (-12.5, -4.3) |
| **Demographic factors** |  |  |  |  |  |  |  |  |
| Women’s age |  |  |  |  |  |  |  |  |
| 15-24 years | 291 (23.2) | -3.0 (-9.4, 3.3) | 114 (20.1) | 2.1 (-4.3, 8.4) | 385 (22.2) | -2.8 (-7.4, 1.7) | 255 (19.4) | -3.8 (-8.3, 0.7) |
| 25-34 years | 127 (18.5) | 0.01 (-6.9, 0.06) | 53 (18.6) | -1.2 (-7.6, 5.2) | 190 (17.3) | -5.6 (-11.0, -0.02) | 124 (11.7) | -6.8 (-11.9, -1.7) |
| 35-49 years | 174 (28.4) | -12.2 (-19.7, -4.8) | 41 (16.1) | 2.9 (-4.5, 10.4) | 141 (19.0) | -8.1 (-14.8, -1.5) | 80 (10.9) | -17.5 (-24.1, -10.9) |
| Parity |  |  |  |  |  |  |  |  |
| None | 285 (23.0) | -2.4 (-8.2, 3.4) | 120 (20.6) | 1.5 (-4.6, 7.6) | 382 (22.1) | -3.2 (-8.0, 1.7) | 288 (18.9) | -4.1 (-8.5, 0.4) |
| 1-4 children | 188 (21.4) | -6.1 (-12.7, 0.5) | 59 (15.3) | 1.5 (-5.0, 7.9) | 245 (16.8) | -5.8 9-10.4, -1.1) | 144 (11.0) | -10.4 (-15.2, -5.6) |
| 5+ children | 120 (27.1) | -6.0 (-16.4, 4.4) | 29 (21.1) | 2.2 (-7.6, 12.1) | 90 (23.3) | -13.5 (-21.3, -5.7) | 28 (9.9) | -17.3 (-25.7, -8.8) |
| **Behavioural factors** |  |  |  |  |  |  |  |  |
| Listening radio |  |  |  |  |  |  |  |  |
| No | 165 (26.2) | -5.4 (-13.9, 3.1) | 44 (20.9) | 4.6 (-3.9, 13.1) | 216 (25.5) | -8.7 (-14.6, -2.7) | 228 (16.8) | -9.4 (-15.3, -3.5) |
| Yes | 427 (22.2) | -3.8 (-8.9, 1.2) | 165 (18.4) | 0.01 (-4.8, 4.8) | 499 (18.4) | -5.2 (-9.1, -1.3) | 231 (13.2) | -9.0 (-13.2, -4.8) |
| Read magazine |  |  |  |  |  |  |  |  |
| No | 354 (22.9) | -3.0 (-4.9, 6.8) | 105 (19.9) | 0.9 (-4.9, 6.8) | 411 (20.9) | -6.6 (-11.5, -1.8) | 295 (14.2) | -8.7 (-12.9, -4.6) |
| Yes | 239 (23.5) | -5.8 (-12.8, 1.2) | 102 (17.7) | 1.4 (-5.2, 8.0) | 304 (19.2) | -3.2 (-7.6, 1.1) | 164 (15.9) | -7.6 (-12.6, -2.7) |
| Watch television |  |  |  |  |  |  |  |  |
| No | 305 (25.2) | -1.0 (-7.7, 5.8) | 71 (24.3) | -13.9 (-8.6, 5.8) | 168 (22.9) | -8.1 (-15.1, -1.1) | 105 (14.7) | -10.5 (-17.0, -3.9) |
| Yes | 288 (21.4) | -4.5 (-9.9, 0.9) | 138 (16.9) | 2.5 (-2.5, 7.5) | 548 (19.3) | -4.5 (-8.2, -0.9) | 354 (14.8) | -6.5 (-10.7, -2.5) |
| **Community-level factors** |  |  |  |  |  |  |  |  |
| Region of residence |  |  |  |  |  |  |  |  |
| Tigray | 73 (33.1) | -1.3 (-11.6, 9.0) | 28 (31.8) | -3.4 (-14.2, 7.5) | 79 (28.4) | -6.5 (-15.3, 2.3) | 55 (21.9) | -11.1 (-19.3, -3.1) |
| Afar | 5 (18.4) | -4.3 (-13.0, 4.4) | 2 914.2) | 17.0 (6.2, 27.9) | 11 (31.2) | -7.4 (-16.8, 1.9) | 8 (23.7) | 5.3 (-1.5, 12.1) |
| Amhara | 139 (31.3) | -13.2 (-25.0, -1.3) | 32 (18.2) | 6.7 (-7.6, 21.1) | 219 (24.9) | -10.4 (-23.3, 2.4) | 95 (14.5) | -16.8 (-26.8, -6.8) |
| Oromia | 163 (20.1) | -0.9 (-11.4, 9.6) | 65 (19.2) | 0.6 (-8.7, 10.0) | 169 (19.8) | -3.0 (-8.4, 2.4) | 130 (16.8) | -3.3 (-10.4, 3.9) |
| Somali | 25 (52.2) | -29.5 (-49.0, -9.9) | 9 (22.7) | -2.6 (-14.3, 9.1) | 23 (20.1) | -2.0 9-11.8, 7.9) | 12 (18.1) | -34 (-5.3, -15.6) |
| Benishangul | 6 (42.7) | -7.1 (-23.4, 9.2) | 2 (35.6) | -12.9 (-30.4, 4.5) | 8 (22.6) | -10.3 9-20.7, 0.1) | 3 (12.3) | -30.3 (-38.6, -22.0) |
| SNNPR** | 44 (17.4) | -1.1 (-12.3, 10.0) | 15 (16.2) | -1.3 (-10.6, 8.0) | 71 (14.9) | -8.0 (-15.0, -1.0) | 25 (7.0) | -10.4 (-19.7, -1.1) |
| Gambella | 4 (31.0) | -3.3 (-28.5, 21.9) | 1 (16.2) | -0.5 (-21.8, 20.9) | 5 (27.2) | 1.7 (-13.1, 16.6) | 5 (28.9) | -2.1 (-22.1, 17.9) |
| Metropolis | 134 (18.4) | -2.9 (-6.0, 0.3) | 55 (15.6) | -0.7 (-3.8, 2.4) | 131 (14.9) | -1.2 (-3.7, 1.3) | 125 (13.6) | -4.8 (-7.4, -2.1) |

**n (%): weighted count and proportion for each variable**

***Diff indicates the point percentage change in prevalence of underweight between 2000 to 2016**

****SNNPR: Southern Nations Nationalities and Peoples Region**
