# Supplementary material for: Trends and determinants of underweight and overweight/obesity among urban Ethiopian women from 2000 to 2016
Source: BMC Public Health. 2020 Aug 24;20:1276. doi: 10.1186/s12889-020-09345-6 (PMC7447570; doi:10.1186/s12889-020-09345-6)
Supplement: Supplementary file 3 — Additional file 3. Percentage point change in the prevalence of overweight/obesity by study factors, 2000–2016. n (%): weighted count and proportion for each variable. *Diff indicates the point percentage change in prevalence of underweight between 2000 to 2016. [file 12889_2020_9345_MOESM3_ESM.docx]

**Additional file 3**

**Percentage point change in the prevalence of overweight/obesity by study factors, 2000–2016**

| **Variables** | **2000** | **2000–2005** | **2005** | **2005–2011** | **2011** | **2011–2016** | **2016** | **2000–2016** |
| --- | --- | --- | --- | --- | --- | --- | --- | --- |
|  | **n (%)** | ***Diff (95% CI)** | **n (%)** | ***Diff (95% CI)** | **n (%)** | ***Diff (95% CI)** | **n (%)** | ***Diff (95% CI)** |
| **Socioeconomic factors** |  |  |  |  |  |  |  |  |
| Women’s education |  |  |  |  |  |  |  |  |
| No schooling | 64 (7.4) | 4.1 (-1.9, 10.2) | 30 (11.5) | 2.1 (-5.1, 9.4) | 105 (13.7) | 4.9 (-1.6, 11.3) | 95 (18.5) | 11.1 (6.1, 16.2) |
| Primary school | 64 (10.3) | 0.3 (-6.2, 6.7) | 30 (10.6) | 2.5 (-3.0, 7.9) | 201 (13.1) | 8.6 (3.4, 13.8) | 228 (21.7) | 11.3 (5.1, 17.6) |
| Secondary and higher | 151 (14.2) | 3.0 (-1.6, 7.6) | 98 (17.2) | 0.8(-4.5, 6.0) | 228 (18.0) | 4.2 (-1.5, 9.9) | 341 (22.2) | 8.0 (3.1, 12.9) |
| Women’s employment |  |  |  |  |  |  |  |  |
| No employment | 109 (9.8) | 2.2 (-1.5, 5.8) | 76 (12.0) | 1.7 (-2.4, 5.8) | 206 (13.7) | 3.4 (-1.4, 8.2) | 219 (17.1) | 7.3 (2.9, 11.6) |
| Formal employment | 133 (14.3) | 6.7 (-1.1, 14.5) | 75 (21.1) | -3.1 (-11.2, 5.0) | 258 (18.0) | 9.2 (2.9, 15.5) | 391 (27.2) | 12.8 (7.1, 18.6) |
| Informal employment | 37 (7.4) | -2.4 (-7.8, 3.0) | 6 (5.0) | 6.2 (0.01, 12.3) | 68 (11.2) | 3.1 (-4.0, 10.2) | 54 (14.3) | 6.9 (3.5, 13.4) |
| Marital status |  |  |  |  |  |  |  |  |
| Not married | 88 (8.1) | 0.2 (-3.4, 3.9) | 43 (8.4) | -0.9 (-4.3, 2.4) | 113 (7.4) | 3.8 (0.6, 7.1) | 143 (11.2) | 3.1 (-0.4, 6.6) |
| Currently married | 144 (14.4) | 6.7 (-0.3, 13.7) | 85 (21.1) | 1.1 (-6.8, 8.9) | 348 (22.1) | 7.2 (-0.4, 14.8) | 414 (29.4) | 14.9 (8.3, 21.6) |
| Formerly married | 47 (9.9) | 5.6 (-1.8, 13.0) | 29 (15.5) | -0.2 (-8.0m 7.7) | 72 (15.3) | 10.2 (17.3, 18.7) | 107 (25.5) | 15.6 (7.7, 23.9) |
| Household wealth status |  |  |  |  |  |  |  |  |
| Poor | 135 (8.4) | 0.2 (-4.5, 4.5) | 54 (8.8) | -6.0 (-14.0, 5.8) | 3 (2.8) | 0.1 (-3.9, 4.0) | 3 (2.9) | -6.8 (-12.3, -1.3) |
| Middle | 107 (16.6) | 4.6 (-0.2, 10.5) | 54 (21.2) | - | - | - | 1 (2.1) | -10.3 (-16.3, -4.4) |
| Rich | 8 (11.6) | 15.8 (3.9, 45.0) | 30 (27.4) | 12.0 (-3.2, 51.8) | 531 (15.4) | 7.0 (2.4, 11.5) | 661 (22.4) | 11.8 (7.5, 16.2) |
| Toilet facility |  |  |  |  |  |  |  |  |
| Unimproved | 46 (6.2) | 3.4 (-1.0, 7.8) | 50 (9.6) | 0.4 (-4.2, 5.0) | 205 (10.0) | 4.3 (-0.7, 9.2) | 216 (14.2) | 8.0 (3.3, 12.7) |
| Improved | 233 (12.8) | 5.2 (0.4, 10.0) | 102 (18.1) | 3.7 (-1.7, 9.1) | 325 (21.7) | 6.7 (1.1, 12.2) | 437 (28.4) | 15.6 (10.6, 20.5) |
| Source of drinking water |  |  |  |  |  |  |  |  |
| Unimproved | 33 (9.1) | -4.8 (-12.6, 3.1) | 4 (4.3) | 7.9 (0.4, 15.3) | 37 (12.1) | 0.1 (-7.0, 7.1) | 66 (12.2) | 3.1 (-4.3, 10.6) |
| Improved | 246 (11.2) | 3.8 (-0.3, 7.8) | 154 (15.0) | 0.2 (-4.2, 4.6) | 496 (15.2) | 8.1 (3.3, 13.0) | 599 (23.4) | 12.1 (7.7, 16.6) |
| **Demographic factors** |  |  |  |  |  |  |  |  |
| Women’s age |  |  |  |  |  |  |  |  |
| 15-24 years | 85 (6.8) | 0.7 (-2.6, 3.9) | 42 (7.4) | -0.1 (-3.4, 3.3) | 128 (7.4) | 2.6 (-0.1, 6.0) | 131 (10.0) | 3.2 (-0.1, 6.5) |
| 25-34 years | 93 (13.6) | 6.4 (0.01, 12.7) | 57 (19.9) | -1.9 (-8.8, 5.0) | 197 (18.0) | 6.6 (0.5, 12.7) | 260 (24.6) | 11.1 (5.7, 16.4) |
| 35-49 years | 101 (16.5) | 6.2 (-1.8, 14.2) | 58 (22.7) | 5.4 (-3.7, 14.5) | 29 (28.1) | 9.3 (-0.9, 19.5) | 274 (37.4) | 20.9 (11.8, 30.1) |
| Parity |  |  |  |  |  |  |  |  |
| None | 105 (8.5) | 1.3 (-2.5, 5.0) | 57 (9.7) | -1.3 (-5.1, 2.5) | 145 (8.4) | 4.2 (0.5, 7.9) | 191 (12.6) | 4.1 (0.5, 7.7) |
| 1-4 children | 115 (13.2) | 6.3 (0.6, 12.0) | 76 (19.5) | 1.8 (-5.0, 8.6) | 309 (21.2) | 10.0 (2.8, 17.3) | 408 (31.3) | 18.1 (12.0, 24.2) |
| 5+ children | 59 (13.3) | 5.0 (-4.3, 14.4) | 25 (18.3) | 2.2 (-7.8, 12.3) | 79 (20.5) | 2.7 (-8.1, 13.6) | 65 (23.3) | 10.0 (-0.2, 20.2) |
| **Behavioural factors** |  |  |  |  |  |  |  |  |
| Listening radio |  |  |  |  |  |  |  |  |
| No | 49 (7.8) | 8.1 (1.2, 14.9) | 33 (15.9) | -0.5 (-8.2, 7.1) | 130 (15.4) | 3.9 (-2.5, 10.3) | 261 (19.3) | 11.5 (6.1, 16.8) |
| Yes | 230 (12.0) | 1.6 (-2.5, 5.7) | 122 (13.6) | 1.3 (-3.2, 5.7) | 403 (14.9) | 8.2 (3.5, 12.9) | 403 (23.0) | 11.1 (6.8, 15.4) |
| Read magazine |  |  |  |  |  |  |  |  |
| No | 157 (10.1) | 3.0 (-2.4, 8.4) | 70 (13.2) | 1.2 (-4.5, 6.8) | 282 (14.3) | 4.1 (-0.7, 8.9) | 382 (18.4) | 8.3 (3.9, 12.6) |
| Yes | 122 (12.1) | 3.3 (-1.0, 7.5) | 88 (15.3) | 0.5 (-4.3, 5.2) | 251 (15.8) | 11.6 (6.3, 16.9) | 283 (27.4) | 15.3 (10.5, 20.2) |
| Watch television |  |  |  |  |  |  |  |  |
| No | 87 (7.2) | 2.7 (-2.3, 7.7) | 29 (9.9) | -2.2 (-7.7, 3.3) | 56 (7.6) | 2.5 (-2.8, 7.8) | 72 (10.1) | 3.0 (-1.7, 7.6) |
| Yes | 192 (14.3) | 1.5 (-3.1, 6.0) | 129 (15.7) | 1.1 (-3.5, 5.8) | 477 (16.9) | 7.9 (3.2, 12.7) | 592 (24.8) | 10.5 (6.0, 15.1) |
| **Community-level factors** |  |  |  |  |  |  |  |  |
| Region of residence |  |  |  |  |  |  |  |  |
| Tigray | 6 (2.7) | 1.9 (-2.6, 6.3) | 4 (4.5) | 4.3 (2.3, 10.8) | 24 (8.8) | 7.3 (-0.5, 15.1) | 41 (16.1) | 13.4 (7.3, 19.6) |
| Afar | 4 (12.5) | 5.8 (-9.3, 20.8) | 2 (18.3) | -8.2 (-23.5, 7.2) | 4 (10.1) | 8.9 (-0.9, 18.8) | 6 (19.0) | 6.5 (-2.8, 15.9) |
| Amhara | 34 (7.5) | 3.7 (-5.7, 13.1) | 20 (11.3) | -2.8 (-12.7, 7.1) | 74 (8.5) | 2.3 (-4.1, 8.6) | 71 (10.7) | 3.2 (-2.2, 8.6) |
| Oromia | 87 (10.7) | 3.5 (-6.1, 13.1) | 48 (14.2) | -0.01 (-10.7, 10.6) | 121 (14.2) | 10.9 (-1.7, 23.5) | 194 (25.1) | 14.3 (2.5, 26.1) |
| Somali | 4 (8.3) | 17.8 (9.2, 26.5) | 11 (26.1) | 6.8 (-11.6, 12.9) | 31 (26.8) | -1.8 9-14.3, 10.6) | 17 (25.0) | 16.7 (7.7, 25.6) |
| Benishangul | 1 (2.9) | 7.9 (-4.1, 19.9) | 1 (10.8) | -1.8 (-15.5, 11.8) | 3 (9.0) | 11.5 (2.3, 20.7) | 5 (20.5) | 17.6 (11.1, 24.1) |
| SNNPR** | 27 (10.7) | 0.03 (-12.3, 12.3) | 10 (10.8) | 9.7 (-1.3, 20.7) | 97 (20.5) | -3.9 (-16.3, 8.5) | 60 (16.5) | 5.8 (-7.8, 19.4) |
| Gambella | 1 (3.8) | 1.4 (-3.9, 6.8) | 1 (5.2) | 8.4 (-0.1, 16.8) | 3 (13.6) | -0.1 (-8.7, 8.4) | 2 (13.5) | 9.7 (4.2, 15.1) |
| Metropolis | 117 (16.1) | 1.5 (-1.9, 4.8) | 62 (17.6) | 2.5 (-1.0, 6.1) | 177 (20.1) | 9.3 (6.4, 12.3) | 269 (29.4) | 13.3 (10.5, 16.0) |

**n (%): weighted count and proportion for each variable**

***Diff indicates the point percentage change in prevalence of underweight between 2000 to 2016**

****SNNPR: Southern Nations Nationalities and Peoples Region**
