# Supplementary figures and images for: Trends and determinants of underweight and overweight/obesity among urban Ethiopian women from 2000 to 2016
Source: BMC Public Health. 2020 Aug 24;20:1276. doi: 10.1186/s12889-020-09345-6 (PMC7447570; doi:10.1186/s12889-020-09345-6)

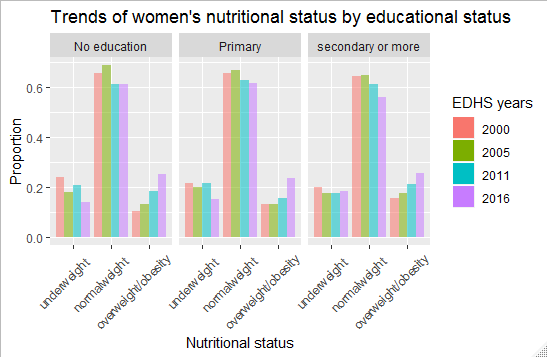


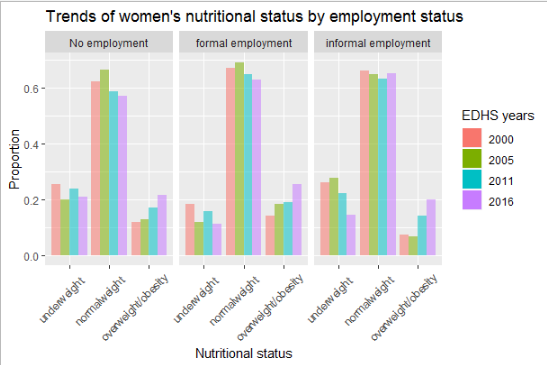


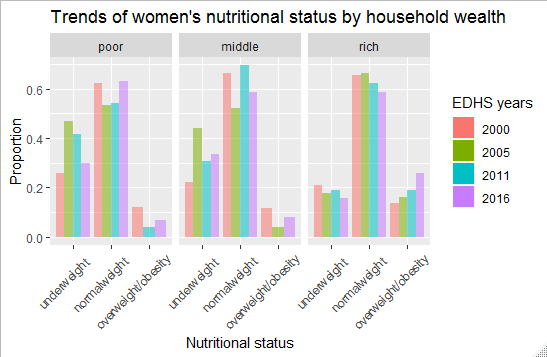


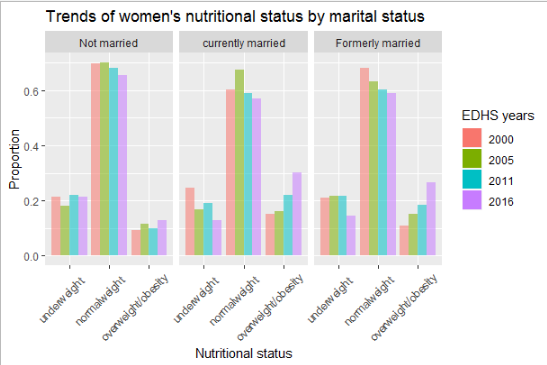


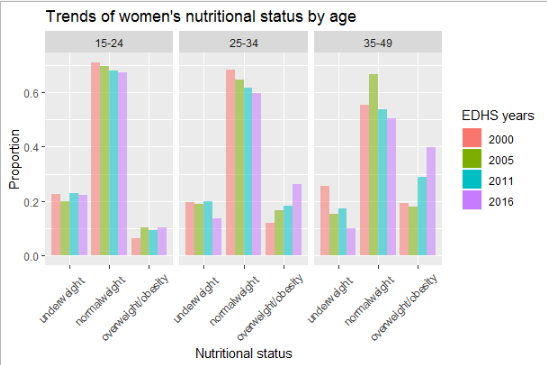


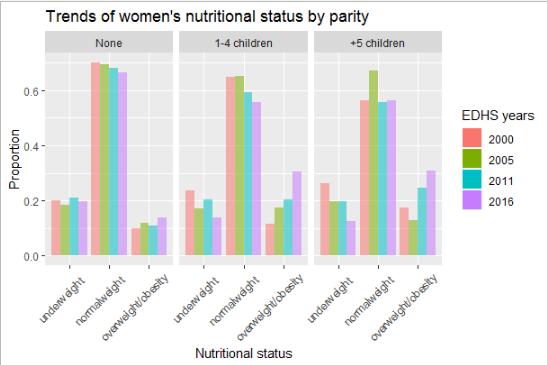


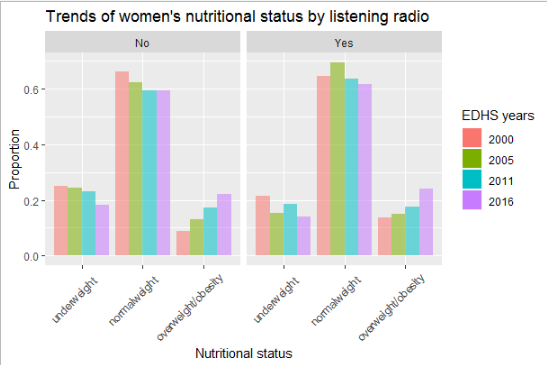


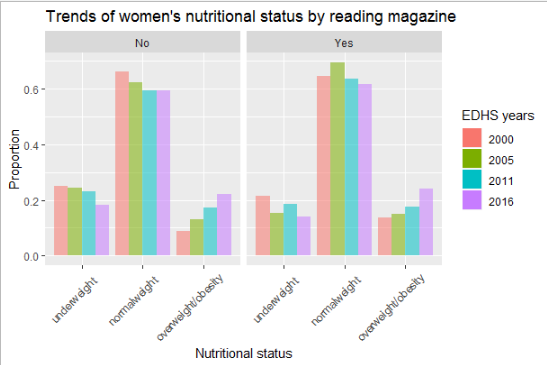


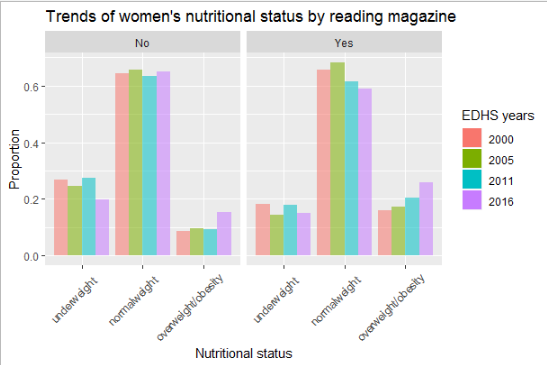


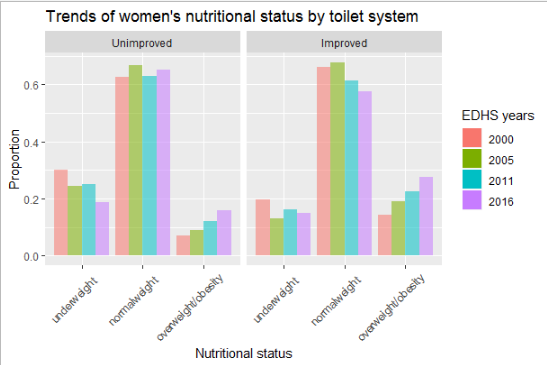


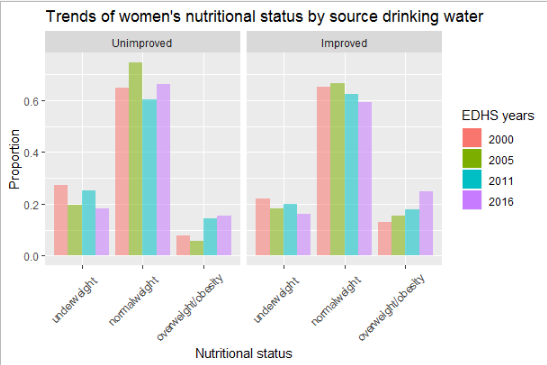

Supplement: Supplementary file 4 — Additional file 4. Bar graphs showing trends of underweight and overweight/obesity by each study variables from 2000 to 2016. [file 12889_2020_9345_MOESM4_ESM.docx]
